# Development and validation of a nomogram for predicting the risk of shoulder-hand syndrome after ischemic stroke: a retrospective study

**DOI:** 10.3389/fnagi.2026.1755994

**Published:** 2026-03-30

**Authors:** Yuan Luo, Yujie Xie, Xin Zeng, Pan Huang, Yong Tang, Akira Miyamoto, Fan Li, Guomin Ding, Guoyin Pang, Bin Liang, Peng Liao, Huanhuan Cao, Xin Tang, Chi Zhang

**Affiliations:** 1 Rehabilitation Medicine Department, The Affiliated Hospital of Southwest Medical University, Luzhou, China; 2 Southwest Medical University, Luzhou, China; 3 Rehabilitation Medicine Department, The First People's Hospital of Neijiang, Neijiang, China; 4 The School of Nursing of The Hong Kong Polytechnic University, Hung Hom, Hong Kong SAR, China; 5 School of Computer Science and Engineering, University of Electronic Science and Technology of China, Chengdu, China; 6 Faculty of Rehabilitation, Nishikyushu University, Saitama, Japan; 7 College of Artificial Intelligence (CUIT Shuangliu Industrial College), Chengdu University of Information Technology, Chengdu, China; 8 The Institute of Digital Health and Medical ITA Innovation Industry, Chengdu, China

**Keywords:** ischemic stroke, logistic, nomogram, prediction model, shoulder-hand syndrome

## Abstract

**Objective:**

This study aims to assess the relevant risk factors and construct a nomogram model to assist in the early identification of shoulder-hand syndrome (SHS) after ischemic stroke.

**Method:**

One thousand five hundred and thirty-nine ischemic stroke patients admitted to the First People’s Hospital of Neijiang from September 2021 to August 2025 were selected as the research subjects. After exclusion, 1,211 patients were selected. These patients were divided into the training group (*N* = 848) and the test group (*N* = 363) at a ratio of 7:3. The coefficients of the predictors in the Logistic regression were used to establish a nomogram and to verify its discriminative power, calibration, and clinical practicability.

**Result:**

Multivariate Logistic regression analysis revealed that age, hypertension, alcohol drinking, muscle strength of the affected upper limb, NIHSS, albumin, and total cholesterol were independent risk factors for shoulder-hand syndrome after ischemic stroke. The nomogram model demonstrated reliable predictive efficacy, with an area under the curve (AUC value) of 0.891 (95% CI, 0.853–0.929). Both DCA and CIC confirmed that the nomogram model has strong clinical practical value.

**Conclusion:**

This study developed a novel predictive model for shoulder-hand syndrome after ischemic stroke, providing a valuable tool for the early identification of high-risk patients in clinical settings.

## 1 Introduction

Stroke remains a leading cause of long-term disability and mortality worldwide, imposing a substantial burden on healthcare systems and patients’ families. Given that the incidence of stroke rises sharply with age, understanding the interplay between aging-related biological changes and post-stroke complications is of paramount importance for improving outcomes in the elderly population (Katan and Luft, 2018; Saposnik et al., 2022; Karisik et al., 2025). With advancements in acute stroke management, survival rates have improved, shifting the focus of care toward rehabilitation and the management of post-stroke complications (de Lima et al., 2023; Hammerbeck et al., 2025). Among these complications, Shoulder-Hand Syndrome (SHS), notably a form of Complex Regional Pain Syndrome type I (CRPS-I), is a frequent and debilitating condition typically affecting the upper extremity on the hemiplegic side (Shafiee et al., 2023; Gao et al., 2025).

SHS is characterized by a triad of sensory disturbances, autonomic dysfunction and motor impairments (Dong et al., 2024; Li et al., 2024). The reported incidence of post-stroke SHS varies widely, ranging from 12 to 74%, depending on the diagnostic criteria and the study population (Huang et al., 2023; Kanika et al., 2023). The onset of SHS can severely hinder the rehabilitation process, leading to increased pain, limited range of motion, muscle atrophy, and prolonged functional dependency in activities of daily living (Meng et al., 2025). Consequently, SHS not only diminishes the quality of life for stroke survivors but also significantly increases the economic burden of post-stroke care (Shi et al., 2025).

Early identification and proactive intervention are crucial for mitigating the adverse effects of SHS. Several risk factors have been implicated in its pathogenesis, including motor impairment, sensory deficits, spasticity, subluxation of the shoulder joint, and specific stroke subtypes (Karabegović et al., 2009; Yu et al., 2023). However, in current clinical practice, the diagnosis of SHS is often established after the full manifestation of symptoms, potentially missing the optimal window for preventive strategies (Saha et al., 2021; Louis et al., 2025). While several clinical assessment tools exist (Varenna et al., 2021; Farzad et al., 2022), there is a lack of a simple, quantitative, and individualized tool for precisely predicting an individual patient’s risk of developing SHS during the early stages post-stroke.

A nomogram is a reliable and intuitive statistical tool that integrates multiple independent risk factors to generate a personalized, numerical probability of a clinical event (Yoshida et al., 2025). By providing a graphical representation of a predictive model, nomograms facilitate the direct estimation of risk and have been widely adopted in oncology and other medical fields to aid in clinical decision-making (Lu et al., 2025). The development of a nomogram for predicting SHS could, therefore, serve as a practical instrument for clinicians to identify high-risk patients at an early stage.

Although previous studies have explored individual risk factors for SHS, a comprehensive predictive model that synthesizes these factors into a clinically applicable tool is currently lacking (Xie et al., 2025). Therefore, the primary objective of this retrospective study was to identify the independent risk factors for SHS following a ischemic stroke and to develop and validate a novel nomogram for the individualized prediction of SHS risk. We anticipate that this tool will enable timely and targeted interventions for susceptible individuals, ultimately improving functional outcomes and quality of life after ischemic stroke.

## 2 Materials and methods

### 2.1 Study design and participants

This retrospective clinical study was approved by the Ethics Committee of the First People’s Hospital of Neijiang City and was conducted in accordance with the Helsinki Declaration. We collected data from patients with ischemic stroke who were admitted to the neurology department and rehabilitation department from September 2021 to August 2025, and established a model to predict shoulder-hand syndrome in patients with ischemic stroke. The following inclusion criteria were established: (1) The patients were aged 18 years or above; (2) All patients met the diagnostic criteria for ischemic stroke and had newly developed responsible lesions confirmed by cranial magnetic resonance imaging. Exclusion criteria included: (1) Hemorrhagic cerebral infarction; (2) Brain tumors; (3) Patients containing substantially incomplete data in the required fields; (4) Patients with prior upper limb surgery. The final study cohort comprised 1,211 stroke patients, who were classified into a training set (*n* = 848) and a validation set (*n* = 363) using a 7:3 split. Discrimination was conducted based on the diagnostic criteria published in 2024 (Abd-Elsayed et al., 2024) to determine whether the stroke patients had SHS. According to these criteria, SHS was diagnosed when patients presented with the following clinical features in the hemiplegic upper extremity: (1) continuing pain that is disproportionate to any inciting event; (2) at least one symptom in three of the four following categories: sensory (hyperesthesia or allodynia), vasomotor (temperature asymmetry or skin color changes), sudomotor/edema (edema or sweating asymmetry), and motor/trophic (decreased range of motion, motor dysfunction, or trophic changes); and (3) no other diagnosis that better explains the signs and symptoms. This retrospective study utilized de-identified data and posed no risk to participants; therefore, the requirement for informed consent was waived. To ensure patient confidentiality, the underlying data will not be publicly disclosed.

SHS diagnosis was recorded at any time during the follow-up period, which extended from stroke onset until hospital discharge. The diagnosis of all spinal cord injury syndromes is made by the attending physicians in the neurology department or the rehabilitation medicine department. Each doctor has at least 5 years of clinical experience in post-stroke rehabilitation. To minimize diagnostic differences, the following standardized process was adopted: (1) All suspected cases were evaluated using a structured assessment form based on diagnostic criteria; (2) In cases of uncertainty, another attending physician confirmed the diagnosis; (3) Consensus meetings were held regularly to discuss ambiguous cases and ensure the consistency of diagnosis application.

### 2.2 Clinical data acquisition

All patient data were extracted from the electronic medical record system of our institution. The collected dataset encompassed demographic characteristics, personal medical history, past medical history, admission assessments, and laboratory test results. Demographic information included age and gender. Personal medical history comprised smoking and drinking habits. Patients with a history of hypertension, heart disease, or diabetes were included in the study. Admission assessments involved evaluations of activities of daily living (ADL), NIHSS, Barden score, manual muscle testing (MMT) of the affected upper limb, and modified Rankin Scale (mRS) classification. Laboratory tests, performed within 24 h of admission, included complete blood count parameters (white blood cell count, red blood cell count, hemoglobin, platelet count, neutrophil count, lymphocyte count, and monocyte count), as well as fasting blood glucose, albumin, triglycerides, total cholesterol, high-density lipoprotein (HDL), low-density lipoprotein (LDL), homocysteine, creatinine, and uric acid. In addition, several composite inflammatory indices were calculated and included in the analysis: systemic immune-inflammation index (SII), systemic inflammation response index (SIRI), aggregate index of systemic inflammation (AISI), neutrophil-to-lymphocyte ratio (NLR), platelet-to-lymphocyte ratio (PLR), monocyte-to-lymphocyte ratio (MLR), lymphocyte-to-monocyte ratio (LMR), and neutrophil-to-albumin ratio (NAR). The detailed calculation formulas for these indices are provided in Table 1.

**Table 1:** Comparison of clinical characteristics between the training and validation cohorts.

Characteristic	Training cohort (*n* = 846)	Internal test cohort (*n* = 363)	*p*-value
Age, Mean ± SD	71 ± 12	71 ± 13	0.47
Gender, *n* (%)			0.366
Male	475 (56.1%)	214 (59%)	
Female	371 (43.9%)	149 (41%)	
Hypertension, *n* (%)			0.713
No	303 (35.8%)	126 (34.7%)	
Yes	543 (64.2%)	237 (65.3%)	
Diabetes, *n* (%)			0.473
No	568 (67.1%)	236 (65%)	
Yes	278 (32.9%)	127 (35%)	
Coronary heart disease, *n* (%)			0.137
No	596 (70.4%)	271 (74.7%)	
Yes	250 (29.6%)	92 (25.3%)	
Smoking, *n* (%)			0.454
No	555 (65.6%)	230 (63.4%)	
Yes	291 (34.4%)	133 (36.6%)	
Alcohol drinking, *n* (%)			0.349
No	603 (71.3%)	249 (68.6%)	
Yes	243 (28.7%)	114 (31.4%)	
Location of occlusion, *n* (%)			0.292
Cerebral cortex	419 (49.5%)	182 (50.1%)	
Cerebellum	45 (5.3%)	21 (5.8%)	
Brainstem	83 (9.8%)	45 (12.4%)	
Basal ganglia	173 (20.4%)	76 (20.9%)	
Multiple	126 (14.9%)	39 (10.7%)	
Hemiplegic limbs, *n* (%)			0.593
Left	379 (44.8%)	159 (43.8%)	
Right	411 (48.6%)	174 (47.9%)	
Bilateral	56 (6.6%)	30 (8.3%)	
MMT upper, *n* (%)			0.103
0–3 level	290 (34.3%)	107 (29.5%)	
4–5 level	556 (65.7%)	256 (70.5%)	
ADL, *n* (%)			0.192
≤60	426 (50.4%)	164 (45.2%)	
61–99	352 (41.6%)	162 (44.6%)	
100	68 (8%)	37 (10.2%)	
Braden score, Mean ± SD	18.53 ± 2.76	18.84 ± 2.93	0.087
NIHSS score, *n* (%)			0.08
≥16	127 (15%)	40 (11%)	
5–15	231 (27.3%)	91 (25.1%)	
<5	488 (57.7%)	232 (63.9%)	
MRS, *n* (%)			0.165
0	12 (1.4%)	3 (0.8%)	
1	197 (23.3%)	99 (27.3%)	
2	162 (19.1%)	79 (21.8%)	
3	131 (15.5%)	59 (16.3%)	
4	225 (26.6%)	76 (20.9%)	
5	119 (14.1%)	47 (12.9%)	
Fasting blood glucose, Mean ± SD	7.8 ± 3.6	8 ± 3.7	0.397
White blood cell, Mean ± SD	7.84 ± 3.2	7.75 ± 3.78	0.697
Red blood cell, Mean ± SD	4.38 ± 0.72	4.39 ± 0.73	0.817
Hemoglobin, Mean ± SD	130 ± 21	130 ± 20	0.634
Platelets, Mean ± SD	201 ± 73	199 ± 76	0.739
Neutrophil count, Mean ± SD	5.63 ± 3.14	5.59 ± 3.6	0.87
Lymphocyte count, Mean ± SD	1.47 ± 0.65	1.61 ± 1.78	0.146
Monocyte count, Mean ± SD	0.53 ± 0.24	0.52 ± 0.25	0.685
Albumin, Mean ± SD	39.5 ± 4.6	39.8 ± 4.4	0.361
Triglyceride, Mean ± SD	2.25 ± 2.14	2.24 ± 1.89	0.95
Total Cholesterol, Mean ± SD	4.63 ± 1.4	4.6 ± 1.33	0.712
HDL, Mean ± SD	1.33 ± 0.43	1.31 ± 0.4	0.323
LDL, Mean ± SD	2.84 ± 1.2	2.8 ± 1.04	0.524
Homocysteine, Mean ± SD	15.1 ± 8.2	14.6 ± 8	0.346
Creatinine, Mean ± SD	83 ± 51	80 ± 33	0.128
Uric acid, Mean ± SD	341 ± 116	336 ± 113	0.471
SII, Mean ± SD	1,045 ± 1,280	1,051 ± 1790	0.957
SIRI, Mean ± SD	3.01 ± 5.17	2.95 ± 6.25	0.862
AISI, Mean ± SD	619 ± 1,093	701 ± 3,162	0.627
NLR, Mean ± SD	5.1 ± 5.4	5 ± 5.6	0.806
PLR, Mean ± SD	166 ± 109	165 ± 111	0.854
MLR, Mean ± SD	0.44 ± 0.35	0.42 ± 0.3	0.448
LMR, Mean ± SD	3.22 ± 1.88	3.51 ± 3.89	0.175
NAR, Mean ± SD	0.15 ± 0.09	0.14 ± 0.1	0.632

MMT, Manual Muscle Testing; ADL, Activities of Daily Living; NIHSS, National Institute of Health Stroke Scale; MRS, Modified Rankin Scale; HDL, High-density lipoprotein; LDL, Low-density lipoprotein; SII, (Neutrophil*platelet)/lymphocyte ratio; SIRI, (Neutrophil* monocyte)/lymphocyte ratio; AISI, (Neutrophil*platelet* monocyte)/lymphocyte ratio; NLR, Neutrophil/lymphocyte ratio; PLR, Platelet/lymphocyte ratio; MLR, Monocyte/lymphocyte ratio; LMR, Lymphocyte/monocyte ratio; NAR, Neutrophil/albumin ratio.

### 2.3 Statistical analysis

All statistical analyses were performed using R software (version 4.2.2). Continuous variables were reported as mean ± standard deviation and compared between groups using Student’s *t*-test. Categorical variables were presented as frequencies (percentages), and group comparisons were performed using the chi-square test. All statistical tests were two-sided, with statistical significance defined as *p* < 0.05.

Prior to model development, we examined the pattern and proportion of missing data for each variable. Missingness was observed for several variables, with proportions ranging from 0.17% (Barthel index score) to 18.28% (homocysteine). To address missing data and avoid potential bias associated with complete-case analysis, we performed multiple imputation using chained equations (MICE). Imputation was conducted using the mice package in R (version 4.2.2) under the missing at random (MAR) assumption. A total of 20 imputed datasets were generated, with 10 iterations for each imputation. Continuous variables were imputed using predictive mean matching, and categorical variables were imputed using logistic regression or polytomous regression as appropriate. Sensitivity analyses comparing the distributions of imputed variables with observed data showed no substantive differences, supporting the plausibility of the MAR assumption.

A total of 1,211 participants were randomly allocated into a training set (*n* = 848) and a validation set (*n* = 363) in a 7:3 ratio. The training set was used for model development, and the validation set was reserved for internal validation. To identify predictors of shoulder-hand syndrome in patients with ischemic stroke and mitigate potential multicollinearity among variables, we employed Least Absolute Shrinkage and Selection Operator (LASSO) logistic regression. This method shrinks the coefficients of less influential variables toward zero, thereby enhancing the predictive accuracy and interpretability of the model. The LASSO model was evaluated and optimized via 10-fold cross-validation, during which the dataset was partitioned into ten subsets. The model underwent iterative training and validation across these subsets to assess its performance and determine the optimal tuning parameter (λ). Model performance was visualized across a range of λ values to guide the selection of the optimal penalty term. The value of λ associated with the minimum cross-validation error (“minimal criteria”) was selected, as it represents the model with the best fit to the data. The resulting LASSO logistic regression model identified independent predictors and constituted the basis for constructing a predictive model for the incidence of ischemic stroke-related shoulder-hand syndrome. Finally, a clinical nomogram was developed based on the selected predictor variables to facilitate individualized risk estimation.

Model performance was evaluated using three key indicators: the area under the receiver operating characteristic curve (AUC), calibration curves, and decision curve analysis (DCA). The AUC quantifies the model’s discriminative ability to differentiate between patients and healthy individuals, with higher values indicating better diagnostic accuracy. Calibration curves were plotted to visualize the agreement between predicted probabilities and observed outcomes. These curves were generated using 1,000 bootstrap resamples to enhance accuracy and mitigate overfitting. Better model calibration is reflected by a closer fit of the calibration curve to the ideal line (45-degree line). DCA was applied to estimate the clinical utility of the model by calculating the net benefit across a range of threshold probabilities, thereby offering a clinically oriented assessment of its practical value.

## 3 Results

### 3.1 Study flow diagram

**Figure 1:**
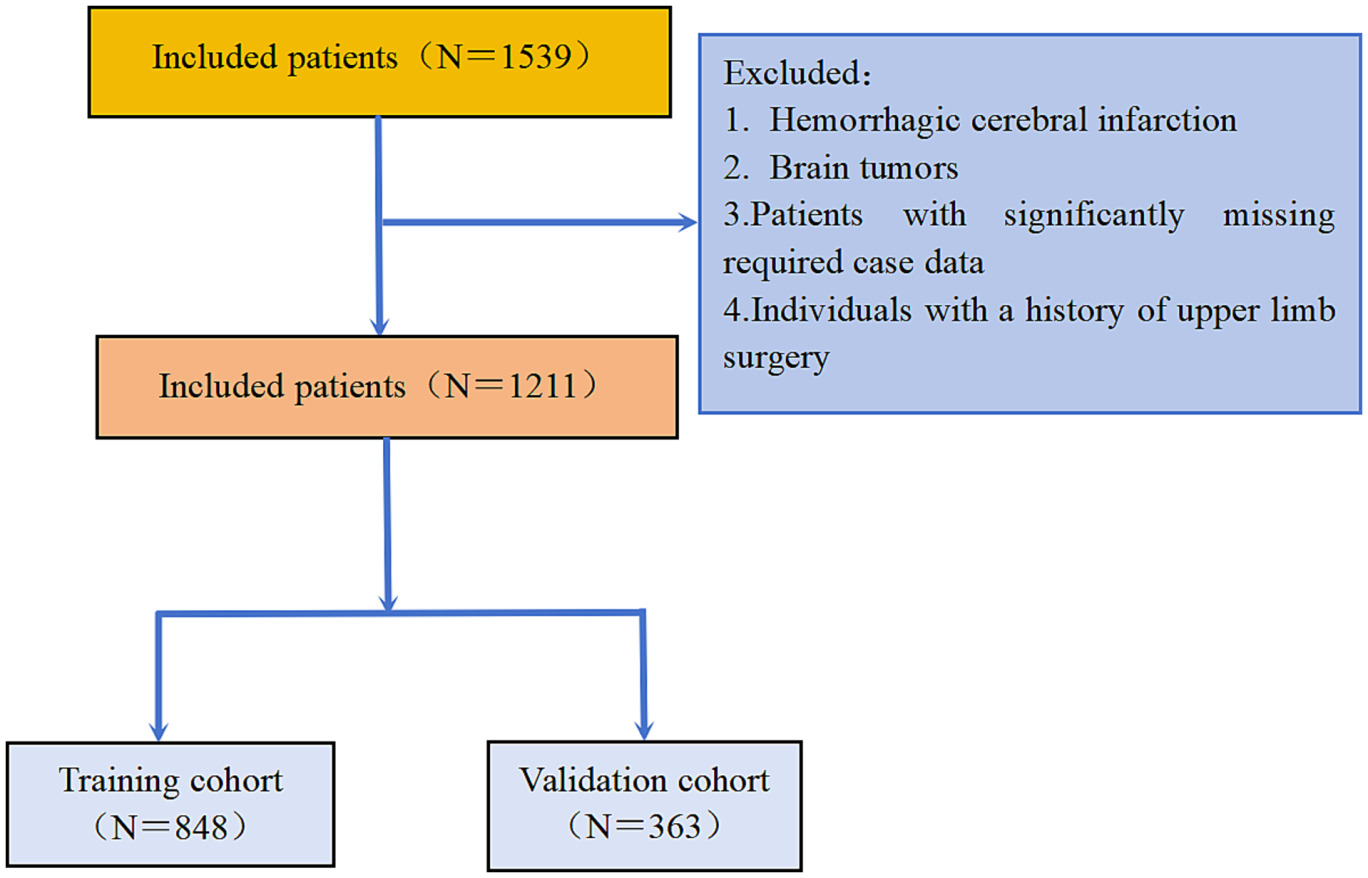
The flowchart of study participants.

Of the 1,539 initially screened stroke patients, 328 were excluded according to the predefined inclusion criteria. Thus, a total of 1,211 participants were ultimately enrolled in the study; the patient selection process is summarized in Figure 1. During data preprocessing, the maximum and minimum values of each variable were reviewed in light of clinical plausibility, and no obvious outliers were identified. The following variables contained missing data, with the percentage of missing values indicated in parentheses: homocysteine (18.28%), fasting blood glucose (15.47%), triglycerides (5.13%), total cholesterol (5.13%), HDL (5.13%), LDL (5.13%), MRS classification (3.97%), NIHSS (2.56%), creatinine (2.56%), uric acid (2.48%), albumin (1.99%), lymphocyte count (0.58%), red blood cells (0.50%), hemoglobin (0.50%), platelets (0.50%), neutrophil count (0.50%), monocyte count (0.50%), white blood cells (0.41%), Barden score (0.33%), and Barthel index score (0.17%). Multiple interpolation methods were employed to address the issue of these missing data.

### 3.2 Patient characteristics

A total of 1,211 patients with ischemic stroke were enrolled in the study and randomly assigned to a training set (*n* = 848) and a validation set (*n* = 363) in a 7:3 ratio. The mean age was 71 ± 12 years in the training set and 71 ± 13 years in the validation set. In the training set, 56.1% of the patients were male, compared with 59.0% in the validation set. Overall, the training and validation sets were well-balanced in terms of all baseline characteristics, with no statistically significant systematic differences observed. A detailed comparison of demographic information, clinical features, relevant scale scores, and laboratory findings between the two groups is provided in Table 1.

The training group used the Wilcoxon test or the chi-square test to compare the various indicators between the SHS group and the non-SHS group. Significant between-group differences were observed in the following variables: age (*p* = 0.006), hypertension (*p* = 0.005), smoking (*p* < 0.001), alcohol drinking (*p* < 0.001), location of occlusion (*p* < 0.001), manual muscle testing (MMT, *p* < 0.001), activities of daily living (ADL, *p* < 0.001), Braden score (*p* < 0.001), NIHSS (*p* < 0.001), hemoglobin (*p* = 0.019), platelets (*p* < 0.001), lymphocyte count (*p* = 0.022), albumin (*p* < 0.001), total cholesterol (*p* < 0.001), HDL (*p* = 0.003), LDL (*p* < 0.001), creatinine (*p* = 0.003), uric acid (*p* = 0.002), and monocyte-to-lymphocyte ratio (MLR, *p* = 0.019). Detailed results are presented in Table 2.

**Table 2:** Patient demographics and baseline characteristics.

Characteristic	Training cohort			Internal test cohort		
	Non-SHS group *N* = 730	SHS group *N* = 116	*P*-value	Non-SHS group *N* = 330	SHS group *N* = 33	*P*-value
Age, Mean ± SD	72 ± 12	68 ± 12	0.006	71 ± 13	67 ± 15	0.081
Gender, *n* (%)			0.76			0.188
Male	408 (55.9%)	67 (57.8%)		191 (57.9%)	23 (69.7%)	
Female	322 (44.1%)	49 (42.2%)		139 (42.1%)	10 (30.3%)	
Hypertension, *n* (%)			0.005			0.013
No	275 (37.7%)	28 (24.1%)		121 (36.7%)	5 (15.2%)	
Yes	455 (62.3%)	88 (75.9%)		209 (63.3%)	28 (84.8%)	
Diabetes, *n* (%)			0.409			0.554
No	494 (67.7%)	74 (63.8%)		213 (64.5%)	23 (69.7%)	
Yes	236 (32.3%)	42 (36.2%)		117 (35.5%)	10 (30.3%)	
Coronary heart disease, *n* (%)			0.349			0.567
No	510 (69.9%)	86 (74.1%)		245 (74.2%)	26 (78.8%)	
Yes	220 (30.1%)	30 (25.9%)		85 (25.8%)	7 (21.2%)	
Smoking, *n* (%)			<0.001			<0.001
No	503 (68.9%)	52 (44.8%)		218 (66.1%)	12 (36.4%)	
Yes	227 (31.1%)	64 (55.2%)		112 (33.9%)	21 (63.6%)	
Alcohol drinking, *n* (%)			<0.001			<0.001
No	548 (75.1%)	55 (47.4%)		236 (71.5%)	13 (39.4%)	
Yes	182 (24.9%)	61 (52.6%)		94 (28.5%)	20 (60.6%)	
Location of occlusion, *n* (%)			<0.001			0.001
Cerebral cortex	373 (51.1%)	46 (39.7%)		169 (51.2%)	13 (39.4%)	
Cerebellum	44 (6%)	1 (0.9%)		21 (6.4%)	0 (0%)	
Brainstem	77 (10.5%)	6 (5.2%)		40 (12.1%)	5 (15.2%)	
Basal ganglia	143 (19.6%)	30 (25.9%)		72 (21.8%)	4 (12.1%)	
Multiple	93 (12.7%)	33 (28.4%)		28 (8.5%)	11 (33.3%)	
Hemiplegic limbs, *n* (%)			0.093			0.175
Left	333 (45.6%)	46 (39.7%)		144 (43.6%)	15 (45.5%)	
Right	345 (47.3%)	66 (56.9%)		156 (47.3%)	18 (54.5%)	
Bilateral	52 (7.1%)	4 (3.4%)		30 (9.1%)	0 (0%)	
MMT upper, *n* (%)			<0.001			<0.001
0–3 level	197 (27%)	93 (80.2%)		78 (23.6%)	29 (87.9%)	
4–5 level	533 (73%)	23 (19.8%)		252 (76.4%)	4 (12.1%)	
ADL, *n* (%)			<0.001			<0.001
≤60	337 (46.2%)	89 (76.7%)		138 (41.8%)	26 (78.8%)	
61–99	326 (44.7%)	26 (22.4%)		157 (47.6%)	5 (15.2%)	
100	67 (9.2%)	1 (0.9%)		35 (10.6%)	2 (6.1%)	
Braden score, Mean ± SD	18.79 ± 2.65	16.86 ± 2.89	<0.001	18.98 ± 2.91	17.36 ± 2.73	<0.001
NIHSS score, *n* (%)			<0.001			<0.001
≥16	57 (7.8%)	70 (60.3%)		21 (6.4%)	19 (57.6%)	
5–15	198 (26.3%)	33 (28.4%)		80 (24.2%)	11 (33.3%)	
<5	475 (65.1%)	13 (11.2%)		229 (69.4%)	3 (9.1%)	
MRS, *n* (%)						<0.001
0	12 (1.6%)	0 (0%)		3 (0.9%)	0 (0%)	
1	192 (26.3%)	5 (4.3%)		98 (29.7%)	1 (3%)	
2	160 (21.9%)	2 (1.7%)		77 (23.3%)	2 (6.1%)	
3	123 (16.8%)	8 (6.9%)		57 (17.3%)	2 (6.1%)	
4	172 (23.6%)	53 (45.7%)		66 (20%)	10 (30.3%)	
5	71 (9.7%)	48 (41.4%)		29 (8.8%)	18 (54.5%)	
Fasting blood glucose, Mean ± SD	7.8 ± 3.7	7.6 ± 2.8	0.378	7.9 ± 3.6	8.5 ± 3.9	0.426
White blood cell, Mean ± SD	7.81 ± 3.15	8.01 ± 3.51	0.566	7.75 ± 3.88	7.75 ± 2.51	0.998
Red blood cell, Mean ± SD	4.39 ± 0.73	4.32 ± 0.62	0.313	4.4 ± 0.74	4.27 ± 0.62	0.267
Hemoglobin, Mean ± SD	130 ± 22	126 ± 18	0.019	131 ± 20	126 ± 17	0.152
Platelets, Mean ± SD	197 ± 72	223 ± 73	<0.001	197 ± 77	222 ± 60	0.031
Neutrophil count, Mean ± SD	5.59 ± 2.94	5.86 ± 4.21	0.511	5.62 ± 3.7	5.3 ± 2.36	0.49
Lymphocyte count, Mean ± SD	1.45 ± 0.66	1.59 ± 0.6	0.022	1.52 ± 1.23	2.49 ± 4.45	0.23
Monocyte count, Mean ± SD	0.53 ± 0.24	0.52 ± 0.22	0.727	0.53 ± 0.25	0.5 ± 0.24	0.583
Albumin, Mean ± SD	39.7 ± 4.7	38.2 ± 4.3	<0.001	39.8 ± 4.4	39.5 ± 3.7	0.399
Triglyceride, Mean ± SD	2.3 ± 2.18	1.94 ± 1.8	0.059	2.24 ± 1.92	2.21 ± 1.55	0.918
Total Cholesterol, Mean ± SD	4.73 ± 1.39	3.97 ± 1.32	<0.001	4.69 ± 1.33	3.71 ± 0.92	<0.001
HDL, Mean ± SD	1.35 ± 0.44	1.24 ± 0.35	0.003	1.32 ± 0.39	1.17 ± 0.4	0.046
LDL, Mean ± SD	2.91 ± 1.22	2.4 ± 0.94	<0.001	2.86 ± 1.05	2.17 ± 0.66	<0.001
Homocysteine, Mean ± SD	15 ± 8	16 ± 9.2	0.259	14.6 ± 8.2	14.5 ± 6.6	0.934
Creatinine, Mean ± SD	85 ± 52	72 ± 42	0.003	80 ± 33	76 ± 32	0.473
Uric acid, Mean ± SD	345 ± 118	314 ± 96	0.002	334 ± 110	354 ± 139	0.409
SII, Mean ± SD	1,028 ± 1,217	1,158 ± 1,621	0.41	1,078 ± 1869	777 ± 499	0.026
SIRI, Mean ± SD	3.07 ± 5.38	2.67 ± 3.6	0.305	3.06 ± 6.53	1.83 ± 1.7	0.009
AISI, Mean ± SD	612 ± 1,092	659 ± 1,102	0.673	730 ± 3,313	409 ± 411	0.102
NLR, Mean ± SD	5.2 ± 5.4	4.7 ± 5.3	0.405	5.2 ± 5.8	3.6 ± 2.3	0.002
PLR, Mean ± SD	166 ± 108	169 ± 113	0.799	167 ± 111	152 ± 114	0.484
MLR, Mean ± SD	0.45 ± 0.36	0.38 ± 0.26	0.019	0.43 ± 0.31	0.32 ± 0.19	0.004
LMR, Mean ± SD	3.18 ± 1.92	3.47 ± 1.63	0.089	3.37 ± 3.52	4.91 ± 6.45	0.187
NAR, Mean ± SD	0.14 ± 0.09	0.16 ± 0.12	0.246	0.14 ± 0.11	0.13 ± 0.06	0.356

MMT, Manual Muscle Testing; ADL, Activities of Daily Living; NIHSS, National Institute of Health Stroke Scale; MRS, Modified Rankin Scale; HDL, High-density lipoprotein; LDL, Low-density lipoprotein; SII, (Neutrophil*platelet)/lymphocyte ratio; SIRI, (Neutrophil* monocyte)/lymphocyte ratio; AISI, (Neutrophil*platelet* monocyte)/lymphocyte ratio; NLR, Neutrophil/lymphocyte ratio; PLR, Platelet/lymphocyte ratio; MLR, Monocyte/lymphocyte ratio; LMR, Lymphocyte/monocyte ratio; NAR, Neutrophil/albumin ratio.

### 3.3 Selection of main predictive factors for SHS after ischemic stroke

**Figure 2:**
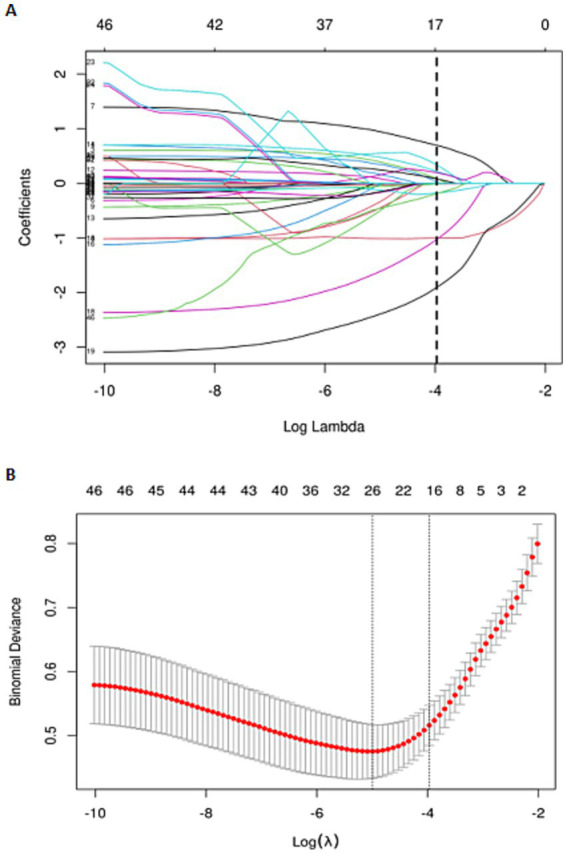
Lasso regression cross-validation plot **(A)** and lasso regression coefficient path plot **(B)**.

**Figure 3:**
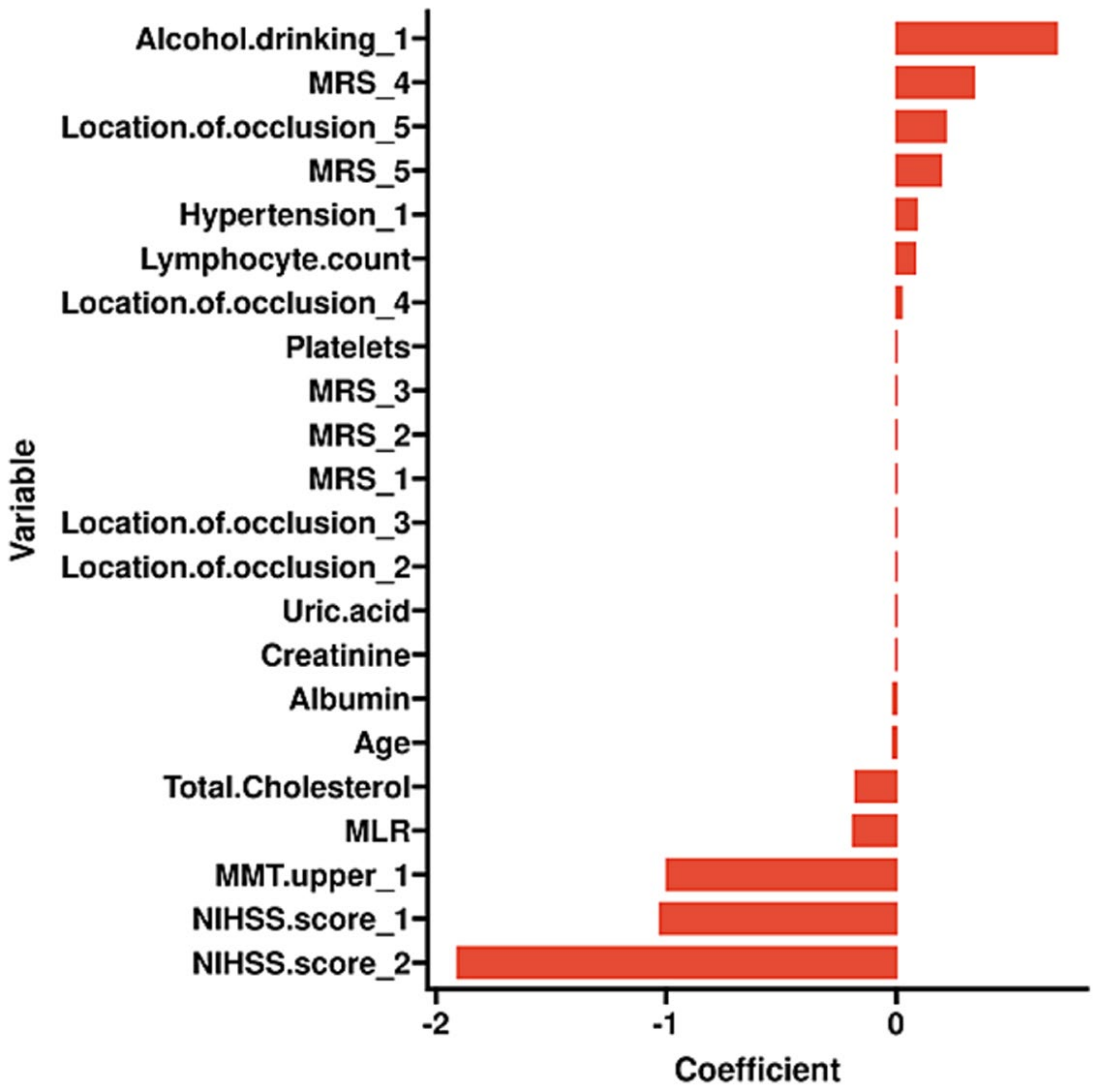
Plot of coefficient profile.

To identify risk factors associated with the development of shoulder-hand syndrome (SHS) in patients with ischemic stroke, we applied Least Absolute Shrinkage and Selection Operator (LASSO) regression analysis to the training set. This method performs feature selection by shrinking the coefficients of less relevant variables to zero, thereby retaining the most influential predictors. By doing so, LASSO regression mitigates overfitting, improves model generalizability, and enhances interpretability. The optimal regularization parameter (λ) was selected through 10-fold cross-validation using the minimum criterion. From an initial set of 38 candidate variables, the LASSO model identified 14 clinically meaningful predictors: age, hypertension, alcohol consumption, lesion location, muscle strength of the affected upper limb, NIHSS, MRS grade, platelet count, lymphocyte count, albumin, total cholesterol, creatinine, uric acid, and monocyte-to-lymphocyte ratio (MLR). The variable selection process is visually summarized in Figures 2A,B. The final regression coefficients are reported in Table 3, and the distribution of these coefficients is illustrated in Figure 3.

**Table 3:** The coefficients of Lasso regression analysis.

Variable	Coefficient
(Intercept)	1.8267675134
Age	−0.0147691659
Gender	0.0000000000
Hypertension	0.0879615205
Diabetes	0.0000000000
Coronary heart disease	0.0000000000
Smoking	0.0000000000
Alcohol drinking	0.6961160297
Location of occlusion (Cerebellum)	0.0000000000
Location of occlusion (Brainstem)	0.0000000000
Location of occlusion (Basal ganglia)	0.0217369791
Location of occlusion (Multiple)	0.2144167692
Hemiplegic limbs	0.0000000000
MMT upper	−0.9973992081
ADL	0.0000000000
Braden score	0.0000000000
NIHSS score (5–15)	−1.027392608
NIHSS score (<5)	−1.905952342
MRS (level 1)	0.0000000000
MRS (level 2)	0.0000000000
MRS (level 3)	0.0000000000
MRS (level 4)	0.3368416317
MRS (level 5)	0.1940901281
Fasting blood glucose	0.0000000000
White blood cell	0.0000000000
Red blood cell	0.0000000000
Hemoglobin	0.0000000000
Platelets	0.0001506844
Neutrophil count	0.0000000000
Lymphocyte count	0.0816577960
Monocyte count	0.0000000000
Albumin	−0.0127721451
Triglyceride	0.0000000000
Total Cholesterol	−0.1773338957
HDL	0.0000000000
LDL	0.0000000000
Homocysteine	0.0000000000
Creatinine	−0.0008412074
Uric acid	−0.0006439205
SII	0.0000000000
SIRI	0.0000000000
AISI	0.0000000000
NLR	0.0000000000
PLR	0.0000000000
MLR	−0.1877942248
LMR	0.0000000000
NAR	0.0000000000

MMT, Manual Muscle Testing; ADL, Activities of Daily Living; NIHSS, National Institute of Health Stroke Scale; MRS, Modified Rankin Scale; HDL, High-density lipoprotein; LDL, Low-density lipoprotein; SII, (Neutrophil*platelet)/lymphocyte ratio; SIRI, (Neutrophil* monocyte)/lymphocyte ratio; AISI, (Neutrophil*platelet* monocyte)/lymphocyte ratio; NLR, Neutrophil/lymphocyte ratio; PLR, Platelet/lymphocyte ratio; MLR, Monocyte/lymphocyte ratio; LMR, Lymphocyte/monocyte ratio; NAR, Neutrophil/albumin ratio.

### 3.4 Construction of a predictive model for SHS in patients with ischemic stroke

**Figure 4:**
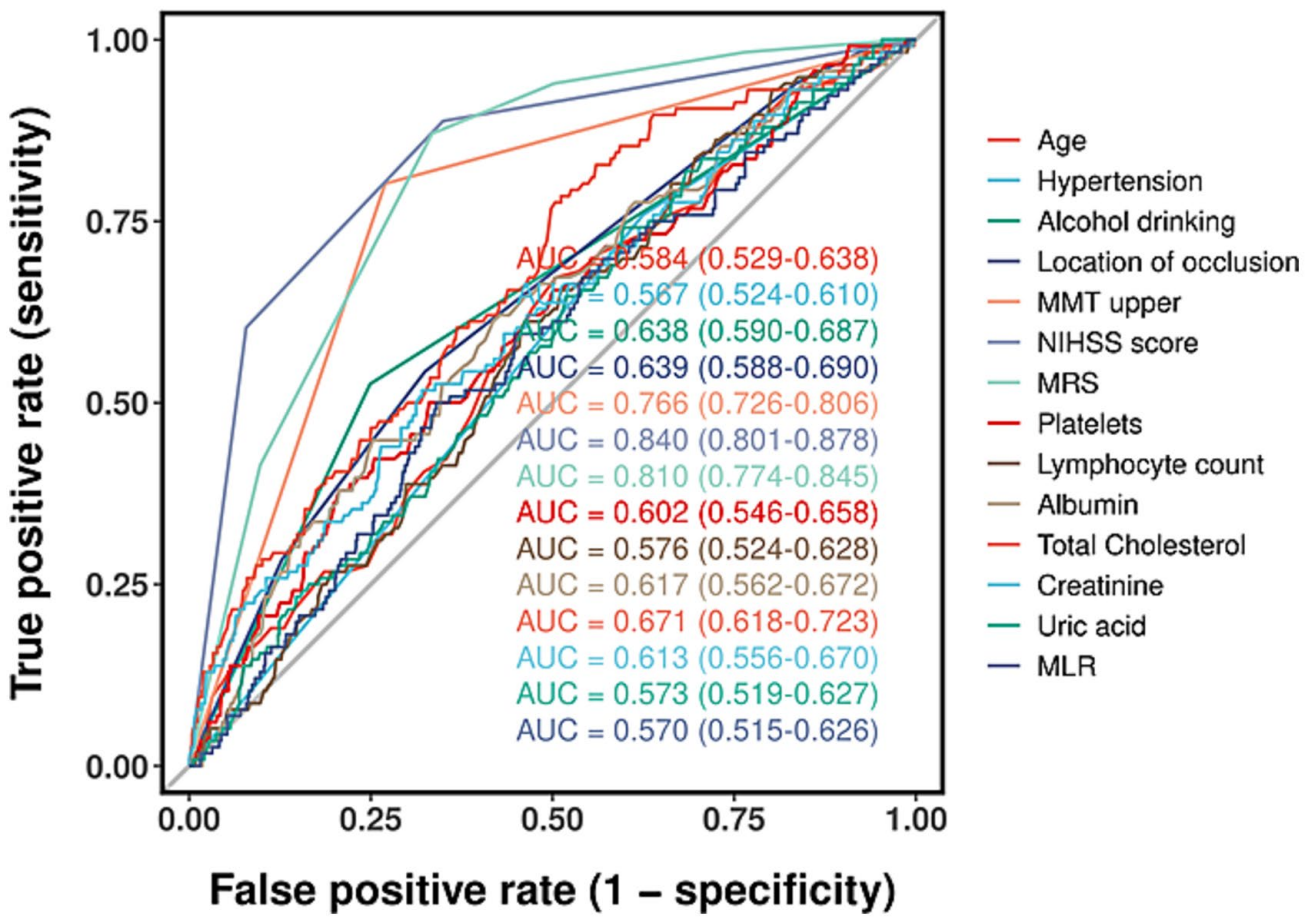
ROC curve analysis 14 candidate diagnostic indicators.

**Figure 5:**
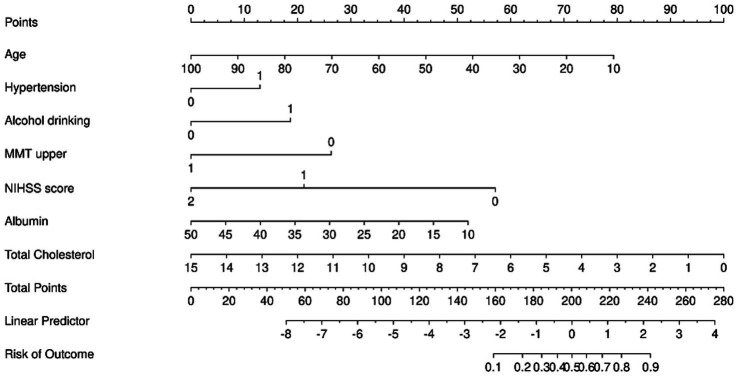
A nomogram for predicting shoulder-hand syndrome in patients with ischemic stroke.

As shown in Figure 4, all variables retained from the feature selection process exhibited area under the curve (AUC) values greater than 0.5. The specific AUC values were as follows: age, 0.584; hypertension, 0.567; alcohol consumption, 0.638; lesion location, 0.639; upper limb manual muscle testing (MMT), 0.766; NIHSS, 0.840; Modified Rankin Scale (MRS), 0.810; platelets, 0.602; lymphocyte count, 0.576; albumin, 0.617; total cholesterol, 0.671; creatinine, 0.613; uric acid, 0.573; and monocyte-to-lymphocyte ratio (MLR), 0.570. To construct a refined prediction model, multivariate logistic regression was performed on the training set. The following non-significant predictors were excluded based on a threshold of *p* < 0.05: location of occlusion (*p* = 0.287), platelets (*p* = 0.521), lymphocyte count (*p* = 0.129), creatinine (*p* = 0.083), uric acid (*p* = 0.094), and MLR (*p* = 0.184) (Table 4). The final model retained seven independent predictors: age, hypertension, alcohol consumption, affected upper limb MMT, NIHSS, albumin, and total cholesterol. These variables were incorporated into a nomogram to estimate the individualized probability of SHS after ischemic stroke (Figure 5).

**Table 4:** Results of multivariate logistic regression for training cohort.

Characteristic	*N*	Event N	OR	95% CI	*P*-value
Age	843	116	0.96	0.93, 0.98	<0.001
Hypertension					
No	301	28	ref	ref	
Yes	542	88	2.08	1.11, 3.93	0.023
Alcohol drinking					
No	601	55	ref	ref	
Yes	242	61	3.42	1.89, 6.17	<0.001
Location of occlusion					
Cerebral cortex	417	46	ref	ref	
Cerebellum	44	1	0.31	0.04, 2.66	0.287
Brainstem	83	6	0.67	0.2, 2.2	0.509
Basal ganglia	173	30	1.74	0.86, 3.51	0.123
Multiple	126	33	2.05	0.98, 4.31	0.057
MMT upper					
0–3 level	289	93	ref	ref	
4–5 level	554	23	0.35	0.17, 0.75	0.007
NIHSS score					
≥16	127	70	ref	ref	
5–15	230	33	0.12	0.06, 0.24	<0.001
<5	486	13	0.06	0.02, 0.15	<0.001
Platelets	843	116	1	1, 1.01	0.521
Lymphocyte count	843	116	1.48	0.89, 2.45	0.129
Albumin	843	116	0.91	0.85, 0.97	0.004
Total Cholesterol	843	116	0.75	0.6, 0.94	0.012
Creatinine	843	116	0.99	0.99, 1	0.083
Uric acid	843	116	1	0.99, 1	0.094
MLR	843	116	0.44	0.13, 1.48	0.184

MMT, Manual Muscle Testing; NIHSS, National Institute of Health Stroke Scale; MLR, Monocyte/lymphocyte ratio.

### 3.5 Validation of the nomogram

**Figure 6:**
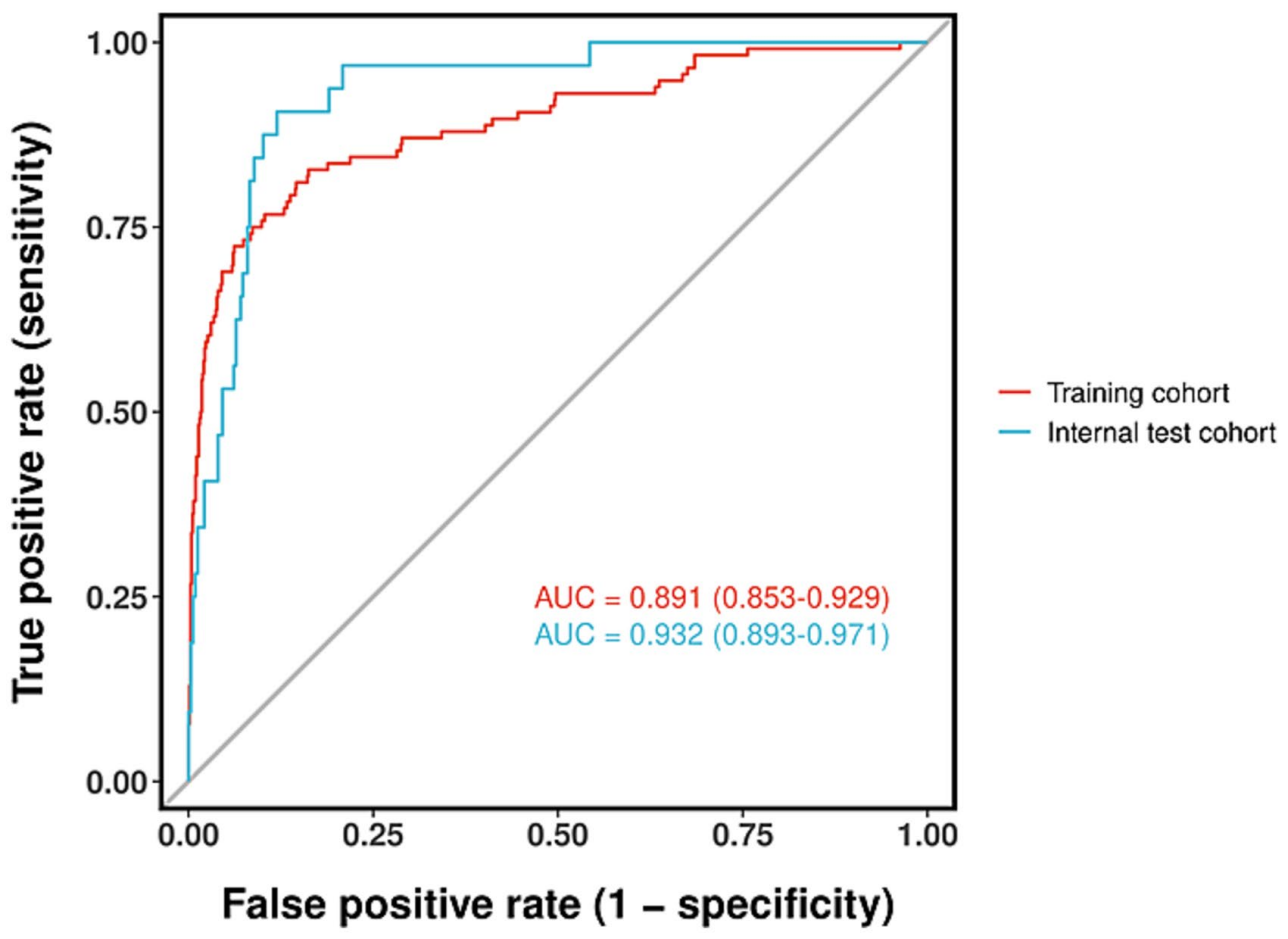
ROC curve for the nomogram based on the training cohort and internal validation cohort.

**Figure 7:**
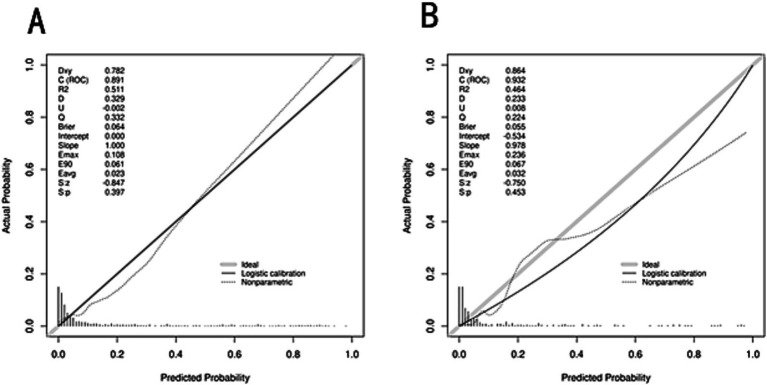
Plots of the calibration curves of the nomogram in the training cohort **(A)** and internal validation cohort **(B)**.

The predictive model performed well. The AUC value of the ROC curve of the training cohort was 0.891 (95% CI, 0.853–0.929). The AUC value of the internal test cohort was 0.932 (95% CI, 0.893–0.971), indicating high accuracy (Figure 6). The Hosmer-Lemesow goodness-of-fit test was used to evaluate the calibration of the prediction model. The calibration curve (Figures 7A,B) shows that there is a strong correlation between the predicted probabilities and the actual occurrence of SHS after ischemic stroke in the validation and training data sets.

### 3.6 Clinical application evaluation of NomoMap

**Figure 8:**
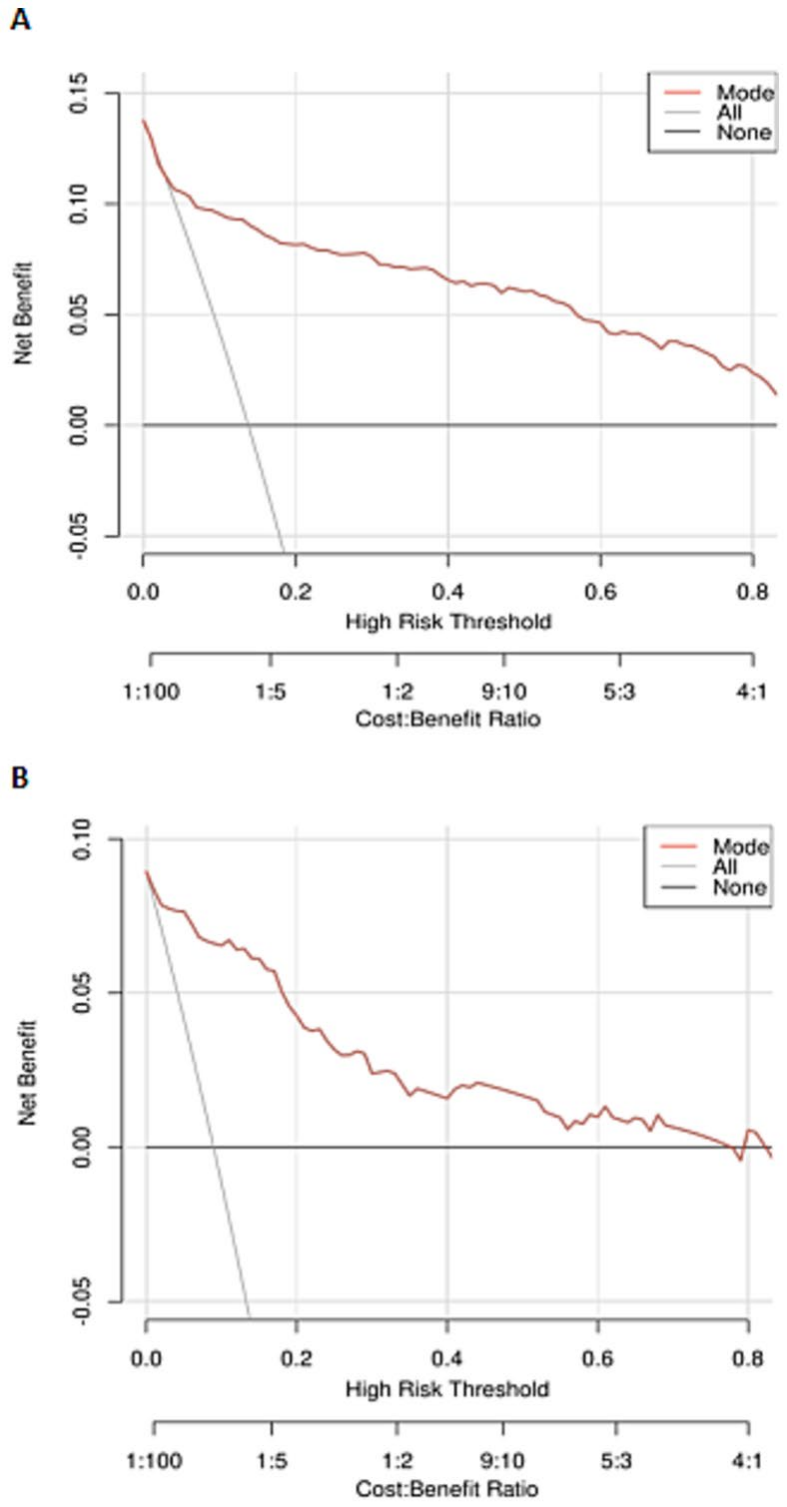
Decision curve analysis (DCA) of the nomogram: **(A)** The training cohort; **(B)** the internal validation cohort.

We further conducted DCA to evaluate the clinical application of the newly developed nomogram for SHS in patients with ischemic stroke. In Figures 8A,B, the nomogram shows a significant net benefit in both the training and validation cohorts. These findings indicate that the newly established nomogram for SHS in patients with ischemic stroke has important clinical practical value.

## 4 Discussion

In this retrospective study, we developed and validated a novel nomogram for the individualized prediction of SHS risk in patients following ischemic stroke. By systematically analyzing a comprehensive set of demographic, clinical, and laboratory variables, we identified seven independent predictors: advanced age, hypertension, alcohol consumption, reduced muscle strength of the affected upper limb, higher NIHSS, lower serum albumin, and elevated total cholesterol. The integration of these factors into a visually accessible nomogram resulted in a model with excellent discriminative ability, as evidenced by AUC values of 0.891 and 0.932 in the training and validation cohorts, respectively. Furthermore, the model demonstrated satisfactory calibration and a significant net benefit upon decision curve analysis, underscoring its robust predictive performance and potential clinical utility for the early identification of high-risk patients.

Several previous studies have explored risk factors for SHS, but few have developed comprehensive, clinically applicable prediction tools. Yu et al. recently developed a random forest-based predictive model for SHS, identifying D-dimer, C-reactive protein, and hemoglobin as the top three important predictors (Yu et al., 2023). Xie et al. presented a nomogram for complex regional pain syndrome after stroke. This model shares some of the same predictive factors with our model, but also includes different variables such as depression and fasting blood glucose. Although their model performed well (with an AUC of 0.867), our logistic regression model has comparable or slightly higher discriminatory ability (with an AUC of 0.891 in the training group and 0.932 in the validation group), and uses different predictive indicators (Xie et al., 2025).

Our findings regarding the independent risk factors are largely consistent with and extend the current understanding of SHS pathophysiology. The strong association between upper limb muscle weakness and SHS risk is well-documented and biologically plausible (Santamato et al., 2010; Kalinowska et al., 2025). Flaccid paralysis or significant weakness in the acute phase of stroke can lead to joint instability, particularly glenohumeral subluxation, and impaired venous and lymphatic drainage due to the loss of the muscle pump effect. This creates a predisposing environment for the development of neurogenic inflammation and pain, which are hallmarks of SHS (Song et al., 2025). Similarly, the NIHSS, a comprehensive measure of stroke severity, emerged as a powerful predictor. A more severe neurological deficit likely reflects a greater extent of central nervous system injury, which may dysregulate the sympathetic nervous system and peripheral inflammatory responses, thereby increasing susceptibility to SHS (You et al., 2024).

The identification of serum albumin and total cholesterol as independent risk factors introduces intriguing pathophysiological considerations. Hypoalbuminemia is a recognized marker of malnutrition and systemic inflammatory status (Heidari et al., 2015). In the context of stroke, low albumin levels may indicate a diminished capacity to counteract the intense oxidative stress and inflammatory cascade that follows cerebral ischemia. This systemic pro-inflammatory state could potentially exacerbate the localized neurogenic inflammation in the affected limb, triggering or amplifying SHS (Hashem et al., 2018). Conversely, the role of hypercholesterolemia is more complex. While traditionally viewed as a vascular risk factor, cholesterol is also a crucial component of cell membranes and is involved in neuronal repair. The observed association with SHS may relate to its role in systemic inflammation or its potential influence on peripheral nerve integrity and pain signaling pathways (Bringeland et al., 2016). However, the exact mechanisms warrant further investigation.

The inclusion of modifiable lifestyle factors, such as alcohol consumption, and comorbidities like hypertension, adds a valuable dimension to our model. Chronic hypertension can cause microvascular damage and impair autoregulation of blood flow, potentially compromising perfusion and increasing susceptibility to tissue injury in the paretic limb (Toyoda et al., 2006; Phuchongchai et al., 2024). Alcohol abuse may contribute through nutritional deficiencies, direct neurotoxicity, or by exacerbating autonomic dysfunction. The association with advanced age aligns with the general observation that tissue resilience and regenerative capacity decline with age, making older patients more vulnerable to post-stroke complications (Cho et al., 2022; Unger et al., 2024). From an aging neuroscience perspective, the strong predictive role of age observed in our model likely reflects the cumulative impact of age-related physiological changes, particularly inflammaging—a state of chronic, low-grade systemic inflammation that accompanies advancing age (Oh et al., 2023). Inflammaging is characterized by elevated levels of pro-inflammatory cytokines (e.g., IL-6, TNF-α) and heightened immune responses, which can exacerbate the neuroinflammatory cascade triggered by cerebral ischemia. This sustained inflammatory milieu may predispose elderly stroke patients to maladaptive peripheral inflammation in the hemiplegic limb, thereby facilitating the development of SHS (Mattson and Arumugam, 2018).

A key strength of our study lies in the rigorous methodology employed for variable selection and model validation. The use of LASSO regression prior to multivariate logistic regression effectively mitigated the risk of overfitting by filtering out redundant variables from a large initial dataset. This approach enhances the model’s generalizability and clinical interpretability. The subsequent development of the nomogram translates a complex statistical model into a user-friendly tool, allowing clinicians to easily calculate a patient’s individualized risk by summing the points assigned to each predictive variable. The high AUC values across both cohorts confirm the model’s strong discriminatory power, surpassing that of any single predictor. The calibration curves indicated good agreement between predicted probabilities and observed outcomes, and the positive net benefit on DCA suggests that using this model to guide clinical decisions would be beneficial across a wide range of threshold probabilities.

This study has several limitations that should be acknowledged. First, its retrospective and single-center design inherently carries risks of selection bias and limits the generalizability of our findings. External validation using prospective, multi-center datasets is essential to confirm the model’s robustness and transportability to other patient populations. Second, despite our efforts to include a wide array of variables, certain potential predictors, such as detailed sensory assessments, spasticity scores, or the presence of shoulder subluxation confirmed by imaging, were not consistently available in our electronic medical records and thus not included in the analysis. Their incorporation in future studies could potentially improve the model’s predictive accuracy. Third, the diagnosis of SHS, while based on contemporary diagnostic criteria, can be subjective and may vary among clinicians. Although we attempted to minimize diagnostic variability through structured assessment forms and consensus meetings, we did not formally assess inter-rater reliability. This represents a potential source of information bias and should be addressed in future prospective studies by incorporating standardized training for assessors and formal reliability testing. Fourth, and importantly, while all patients underwent cranial magnetic resonance imaging to confirm the diagnosis of ischemic stroke, neuroimaging-derived variables—such as lesion location, volume, hemisphere, cortical versus subcortical involvement, and markers of cerebral small vessel disease or brain atrophy—were not systematically quantified and incorporated into our predictive model (Junttola et al., 2021). Finally, as with any predictive model, the nomogram indicates probability, not certainty. It is designed to be an aid to clinical judgment, not a replacement for it.

## 5 Conclusion

In conclusion, we have successfully developed and internally validated a nomogram that integrates seven readily available clinical parameters to predict the risk of SHS after ischemic stroke. This tool demonstrates excellent predictive performance and holds promise for facilitating early risk stratification in clinical practice. By enabling the identification of high-risk individuals during the acute or early subacute phase of stroke, clinicians can prioritize these patients for closer monitoring and early, targeted preventive strategies, such as specialized positioning, careful handling of the hemiplegic limb, and prompt pain management. Future prospective, multi-center studies are warranted to externally validate this model and to investigate whether its implementation in clinical workflows can effectively reduce the incidence and severity of post-stroke SHS, thereby improving long-term functional outcomes and quality of life for stroke survivors.

## Data Availability

The raw data supporting the conclusions of this article will be made available by the authors, without undue reservation.
